# GENDER AND EDUCATIONAL DIFFERENCES IN TRAJECTORIES OF PHYSIOLOGICAL AGEING

**DOI:** 10.1093/geroni/igae098.1221

**Published:** 2024-12-31

**Authors:** Mikaela Bloomberg, Andrew Steptoe

**Affiliations:** University College London, London, England, United Kingdom; University College London, London, England, United Kingdom

## Abstract

Though gender and education are key characteristics that contribute to heterogeneity in ageing-related outcomes, how physiological age (PA) differs by gender and education level is not yet clear. Furthermore, previous studies examining PA are cross-sectional; understanding how PA changes with increasing chronological age is a necessary next step. We examined how gender and education interacted to inform longitudinal PA trajectories using three waves of data (2004/05-2012/13) from 8,891 ELSA participants. PA was derived in a healthy subsample then validated in the full analytic sample by confirming associations between PA and incident ageing-related chronic conditions, memory impairment, and functional limitations. We then used joint models adjusted for birth cohort to produce trajectories of PA between chronological ages 50 and 80 in men and women at three levels of education (less than high school, high school, and above high school). Gender differences in PA were minor at age 50, but PA increased faster in women than in men (p < 0.001); by age 70, women had PA 1.6 years (95% confidence interval=1.0-2.1) older than men. Examination of interactions between gender and education revealed that these gender differences occurred among less educated women only (p interaction=0.03); women educated above high school level maintained PA younger than men at age 70. These results suggest that higher education may be important to reduce gender disparities in physiological ageing. Policies to promote gender equity in higher education may contribute to reducing gender inequalities in a wide range of health outcomes in old age.

